# The UEA sRNA Workbench (version 4.4): a comprehensive suite of tools for analyzing miRNAs and sRNAs

**DOI:** 10.1093/bioinformatics/bty338

**Published:** 2018-05-02

**Authors:** Matthew B Stocks, Irina Mohorianu, Matthew Beckers, Claudia Paicu, Simon Moxon, Joshua Thody, Tamas Dalmay, Vincent Moulton

**Affiliations:** 1 School of Computing Sciences, University of East Anglia, Norwich Research Park, Norwich, UK; 2 School of Biological Sciences, University of East Anglia, Norwich Research Park, Norwich, UK

## Abstract

**Motivation:**

RNA interference, a highly conserved regulatory mechanism, is mediated via small RNAs (sRNA). Recent technical advances enabled the analysis of larger, complex datasets and the investigation of microRNAs and the less known small interfering RNAs. However, the size and intricacy of current data requires a comprehensive set of tools, able to discriminate the patterns from the low-level, noise-like, variation; numerous and varied suggestions from the community represent an invaluable source of ideas for future tools, the ability of the community to contribute to this software is essential.

**Results:**

We present a new version of the UEA sRNA Workbench, reconfigured to allow an easy insertion of new tools/workflows. In its released form, it comprises of a suite of tools in a user-friendly environment, with enhanced capabilities for a comprehensive processing of sRNA-seq data e.g. tools for an accurate prediction of sRNA loci (CoLIde) and miRNA loci (miRCat2), as well as workflows to guide the users through common steps such as quality checking of the input data, normalization of abundances or detection of differential expression represent the first step in sRNA-seq analyses.

**Availability and implementation:**

The UEA sRNA Workbench is available at: http://srna-workbench.cmp.uea.ac.uk. The source code is available at: https://github.com/sRNAworkbenchuea/UEA_sRNA_Workbench

**Supplementary information:**

Supplementary data are available at *Bioinformatics* online.

## 1 Introduction

RNA interference, a highly conserved regulatory mechanism, is mediated via small RNAs (sRNAs). These can be classified into microRNAs (miRNAs) and small interfering RNAs (siRNAs), differentiated by both biogenesis and mode of action (Carthew and Sontheimer, 2009). sRNAs play key roles in gene regulation in eukaryotes (Wilson and Doudna, 2013).

Recent technical advances in high throughput sequencing, in depth and number of available samples and replicates, have enabled the analysis of larger, more complex datasets. Their analysis requires a comprehensive set of tools, able to distinguish patterns from the low-level, noise-like and variation. Drawbacks of existing tools include limited transferability of mRNA-seq methods to sRNA-seq data (Soneson and Delorenzi, 2013), the focus on particular aspects of the analysis e.g. the prediction of miRNAs or the limited number of available analyses within the same suite of tools (Rueda *et al.*, 2015). To address this we have expanded the functionality of the UEA sRNA Workbench (Stocks *et al.*, 2012) by including new features to facilitate its usage on a wide variety of hardware and to enable the seamless linking of its stand-alone components (Beckers *et al.*, 2017). We have reconfigured the code to facilitate an easy future development by members of the s/miRNA community.

## 2 New features and usage

We now describe the main additions to the new version of the Workbench; other, older features are described in (Stocks *et al.*, 2012). We also present features enhancing the usability and versatility of this software, such as the addition of pre-configured templates and availability of the software on the Amazon Web Services (AWS).

### 2.1 Software architecture and usage

The Workbench is implemented in Java, a cross-platform language conferring flexibility between operating systems; all dependencies were compiled per platform forgoing the need for additional configurations. The reconfigured source code is based on a modular design built around a set of interfaces and classes, all of which can be extended. From version 4.4 the source code was released on GitHub.

The Workbench supports and uses a variety of file types from raw data (sequencing files) to processed files. The former category includes *.fastq and/or *.fasta (Cock *et al.*, 2010). Alignment files, generated or accepted within the Workbench, are in PatMaN, SAM (Li *et al.*, 2009) or Bowtie formats (Ziemann *et al.*, 2016), as requested by users of previous versions of the Workbench. Reference sequences (e.g. genomes, transcriptomes) are accepted in *.fasta or indexed formats. Annotation information is currently read as gff files.

Previous versions used traditional data structures for handling sequencing data (raw or indexed). Recently, larger experiments became affordable; the knock-on effect was a proportional increase in the size of raw/processed files, increasing the memory requirements. To reduce the memory footprint we use secondary storage via a relational database. In addition, the main interface was redesigned to facilitate the chaining of tools into workflows. Each node (tool) performs a specific task of either processing or analyzing the data; on node-completion graphical summaries are presented. To configure a workflow, a wizard guides the user through the data input (samples with/without replicates) and reference genome. Upon setup completion, the structure of the project can be inspected using a tree diagram where leaf nodes represent the data files.

Starting with version 4.4 the source code of the UEA sRNA Workbench is available on GitHub, thus enabling the community to amend existing tools and also develop new tools which can be seamlessly linked to the existing framework. To simplify the technical aspects of integrating new features we provide a ‘template’ tool/workflow that already contains the integrative functionality and that can be extended by users.

### 2.2 Improved stand-alone helper tools

The Workbench helper tools were designed to trim the adapters in sRNA-seq data (adapter sequences for versions of Illumina/454 are pre-loaded; custom-made adapters can be specified). A new feature is the ability to process libraries using bespoke adapters for reducing sequencing bias (e.g. high-definition adapters).

The filtering tool was enhanced and added to a stand-alone workflow; it allows the exclusion of unwanted sequences (e.g. tRNA/rRNA fragments, degradation fragments) from multiple datasets as well as the selection of non-transcriptome matching reads. Both tools summarize the output in a size-specific histogram of abundances.

### 2.3 Locus analysis tools

The prediction of sRNA loci is improved by increasing the number and diversity of samples or the sequencing depth. However, a higher number of available reads also requires an approach to determine a signal to noise threshold. Using expression patterns and entropies, two approaches are available in with Workbench, one for the identification of general sRNA loci, **CoLIde** (Mohorianu *et al.*, 2013) and one that improves the prediction of miRNA loci, **miRCat2** (Paicu *et al.*, 2017).

**CoLIde** groups sRNAs in close proximity on the genome that share a common expression pattern into putative loci. The up, down and straight pattern is determined on the relative location of expression intervals and offseted fold change. The expression intervals are either simulated, when no replicates are available, or computed on the normalized, replicated measurements. The patterns can be built on either ordered or un-ordered series. The significance of a locus is based on the dissimilarity of the size class distribution of constituent sRNAs to a random uniform distribution.

**MirCat2** is a tool for miRNA discovery based on a new approach to scan the genome coupled with an entropy approach for the identification of peaks and exclusion of low abundance sequences, below a noise level. First, all putative peaks are identified using a Kullback–Leibler divergence on abundances. The background level is determined by excluding a peak and re-examining the abundance-distribution. Additional, empirical filters include the exclusion of multiple-matching reads, the analysis of local size class distributions as done in CoLIde and the check for miRNA-like variants. The secondary structures of loci (determined using RNALfold) are used for the identification of miRNA loci (Supplementary Fig. S1e and f).

### 2.4 Differential expression analysis of sRNAs

A workflow for processing a sRNA project (consisting of several libraries, with/without replicates) from raw data to the identification of differentially expressed (DE) transcripts and expression patterns (Beckers *et al.*, 2017) is also included. Since this type of analysis may involve a large number of samples, the use of the optimized database feature is default. The workflow comprises of several steps: (i) quality checking of the samples, (ii) evaluation of the effects of various normalizations and selection of an appropriate method, (iii) identification of DE sRNAs and (iv) summarization of expression patterns.

First, diagnostics plots are generated e.g. size class distributions of redundant and non-redundant reads, complemented by complexity analyses (Supplementary Fig. S1a). Additional plots include the nucleotide composition, the Jaccard similarity index (Supplementary Fig. S1c), histograms showing the proportions of read assigned to available annotation classes and scatter and MA plots (Supplementary Fig. S1b). These plots are enhanced by boxplots showing the distribution of differential expression, separated per size class (Supplementary Fig. S1d).

Second, the normalization of expression levels for transcriptome-matching reads is performed. The user can compare the results of up to six normalization procedures [total count normalization, upper quartile, TMM (edgeR), deSeq normalization, quantile adapted for sequencing data and sub-sampling (without replacement) normalization (Mohorianu *et al.*, 2017)]. For each, the quality check plots are recreated allowing the user to select a method that renders the samples most comparable.

Third, DE sRNAs are identified based on fold changes between expression intervals. To exclude low abundance variation, this step uses an offset (noise to signal threshold) calculated using Kullback–Leibler entropy and LOESS smoothing. Lastly, we convert fold-changes into patterns. To assess the DE, users can group sequences sharing a pattern (or motif) and use the sub-sets as starting points for enrichment analyses.

### 2.5 Availability on the AWS

To accommodate the increase in number of samples/replicates and address the difficulty in accessing suitable servers for the analyses, we enabled the use of the Workbench on the AWS via a pre-configured virtual machine, an Amazon Machine Instance. Scripts to facilitate the transfer of samples (via sftp) and the handling the remote connection are also provided. The AWS version of the Workbench allows users to perform analyses on the cloud, allocating and de-allocating resources dynamically and using resources dependent on their requirements and budget.

## 3 Discussion

The latest version of the Workbench enables biologists and bioinformaticians to critically and objectively extract information from larger, more complex sRNA datasets by combining workflows and stand-alone tools complemented with intuitive visualization features.

## Funding

This work was supported by the Biotechnology and Biological Sciences Research Council (grant BBSRC BB/L021269/1 to V.M. and T.D.).

*Conflict of Interest*: none declared.

## Supplementary Material

Supplementary Data

## References

[bty338-B1] Beckers M. et al (2017) Comprehensive processing of high-throughput small RNA sequencing data including quality checking, normalization, and differential expression analysis using the UEA sRNA Workbench. RNA, 23, 823–835. 28289155 10.1261/rna.059360.116 PMC5435855

[bty338-B2] Carthew R.W., Sontheimer E.J. (2009) Origins and mechanisms of miRNAs and siRNAs. Cell, 136, 642–655. 19239886 10.1016/j.cell.2009.01.035 PMC2675692

[bty338-B3] Cock P.J. et al (2010) The Sanger FASTQ file format for sequences with quality scores, and the Solexa/Illumina FASTQ variants. Nucleic Acids Res., 38, 1767–1771. 20015970 10.1093/nar/gkp1137 PMC2847217

[bty338-B4] Li H. et al (2009) The sequence alignment/map format and SAMtools. Bioinformatics, 25, 2078–2079. 19505943 10.1093/bioinformatics/btp352 PMC2723002

[bty338-B5] Mohorianu I. et al (2013) CoLIde: a bioinformatics tool for CO-expression-based small RNA Loci Identification using high-throughput sequencing data. RNA Biol., 10, 1221–1230. 23851377 10.4161/rna.25538 PMC3849171

[bty338-B6] Mohorianu I. et al (2017) Comparison of alternative approaches for analysing multi-level RNA-seq data. PLoS One, 12, e0182694. 28792517 10.1371/journal.pone.0182694 PMC5549751

[bty338-B7] Paicu C. et al (2017) miRCat2: accurate prediction of plant and animal microRNAs from next-generation sequencing datasets. Bioinformatics, 33, 2446–2454. 28407097 10.1093/bioinformatics/btx210 PMC5870699

[bty338-B8] Rueda A. et al (2015) sRNAtoolbox: an integrated collection of small RNA research tools. Nucleic Acids Res., 43, W467–W473. 26019179 10.1093/nar/gkv555 PMC4489306

[bty338-B9] Soneson C., Delorenzi M. (2013) A comparison of methods for differential expression analysis of RNA-seq data. BMC Bioinformatics, 14, 91. 23497356 10.1186/1471-2105-14-91 PMC3608160

[bty338-B10] Stocks M.B. et al (2012) The UEA sRNA workbench: a suite of tools for analysing and visualizing next generation sequencing microRNA and small RNA datasets. Bioinformatics, 28, 2059–2061. 22628521 10.1093/bioinformatics/bts311 PMC3400958

[bty338-B11] Wilson R.C., Doudna J.A. (2013) Molecular mechanisms of RNA interference. Annu. Rev. Biophys., (2013) 42, 217–239. 23654304 10.1146/annurev-biophys-083012-130404 PMC5895182

[bty338-B12] Ziemann M. et al (2016) Evaluation of microRNA alignment techniques. RNA, 22, 1120–1138. 27284164 10.1261/rna.055509.115 PMC4931105

