# Supplementary material for: The UEA sRNA Workbench (version 4.4): a comprehensive suite of tools for analyzing miRNAs and sRNAs
Source: Bioinformatics. 2018 May 2;34(19):3382–4. doi: 10.1093/bioinformatics/bty338 (PMC6157081; doi:10.1093/bioinformatics/bty338)
Supplement: Supplementary Data [file bty338_supp_figs.pdf]

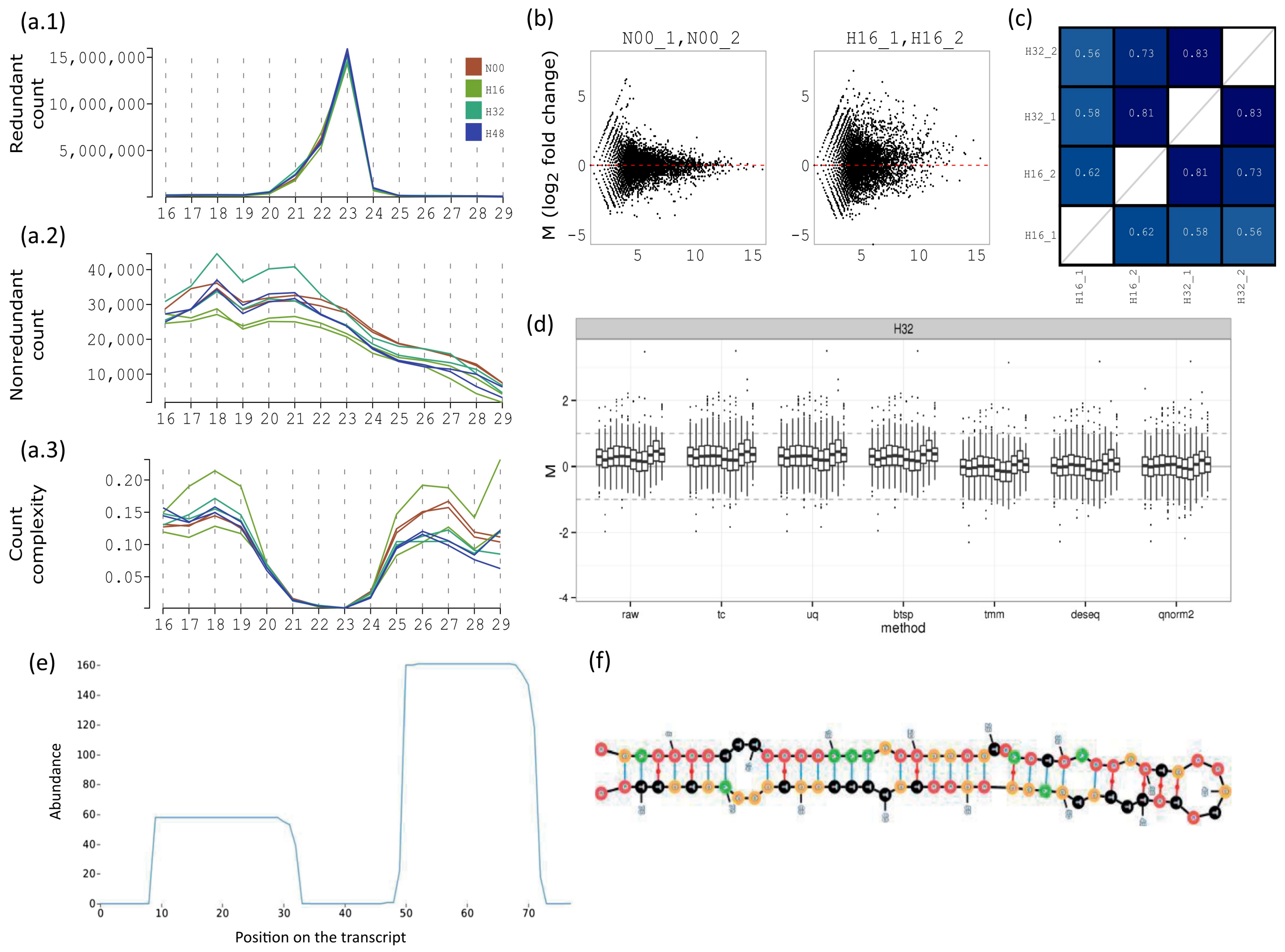

Examples of plots generated with the UEA sRNA Workbench. (a) Diagrams showing the redundant (a1), non-redundant (a2) and complexity (a3) distributions on a *H. sapiens* test data, (b) MA plots comparing biological replicates, used to illustrate two highly reproducible samples (left) and the effect of noise and technical variability (right), (c) Jaccard similarity index computed on two samples with two replicates each showing the higher similarity between replicates than between samples, (d) comparison of several normalization methods based on the distribution of differential expression (DE), separated per size class, post normalization (the raw data and all six normalizations are presented), (e) miRNA presence plot illustrating the typical two-peak pattern (these correspond to the miRNA and miRNA*, respectively), (f) secondary structure of a miRNA locus.
